# Incremental prognostic value of AI-based coronary CT angiography combined with PET/CT dynamic myocardial perfusion imaging in patients with suspected or confirmed coronary artery disease

**DOI:** 10.1186/s12872-026-06217-6

**Published:** 2026-07-16

**Authors:** Ting Wang, Fukai Zhao, Zekun Pang, Jing Ni, Haoran Guo, Yue Chen, Jiao Wang, Jianming Li

**Affiliations:** 1 Clinical School of Cardiovascular Disease, Tianjin Medical University, Tianjin, China; 2 Department of Nuclear Medicine, TEDA International Cardiovascular Hospital, 65 Third Street, Tianjin Economic-Technological Development Area, Tianjin, 300457 China

**Keywords:** Artificial intelligence, AI, Coronary computed tomography angiography, CCTA, Positron emission tomography, PET, Myocardial perfusion imaging, MPI, Coronary artery disease, CAD, Prognosis

## Abstract

**Background:**

Coronary computed tomography angiography (CCTA) and positron emission tomography/computed tomography (PET/CT) myocardial perfusion imaging (MPI) provide complementary anatomical and functional information for coronary artery disease (CAD). However, the prognostic value of integrating CCTA-derived coronary imaging characteristics with PET-MPI parameters remains to be established. Therefore, this retrospective single-center study aimed to evaluate the prognostic and incremental value of integrating artificial intelligence (AI)–based CCTA with PET/CT MPI for predicting major adverse cardiovascular events (MACEs) in patients with CAD.

**Methods:**

Patients with suspected or confirmed CAD underwent PET/CT dynamic MPI and contemporaneous CCTA were retrospectively enrolled and followed for at least 12 months for MACEs. MACEs were defined as the occurrence of at least one of the following endpoints: all-cause death, nonfatal myocardial infarction, new-onset heart failure, rehospitalization for angina, or late coronary revascularization. Clinical characteristics, AI-derived CCTA features, and PET-MPI parameters were collected. To reduce the number of candidate variables and mitigate potential multicollinearity, LASSO regression was applied as an exploratory variable selection method. Variables with non-zero coefficients were subsequently included in multivariable Cox proportional hazards models to assess their associations with the risk of MACEs. Dose-response relationships were further explored using restricted cubic spline analyses. Incremental prognostic value was evaluated using hierarchical Cox regression models.

**Results:**

A total of 71 patients (mean age 60.17 ± 12.36 years; 59.2% male) were included, with 18 MACEs occurring over a median follow-up of 25.69 months. Patients with MACEs more frequently exhibited obstructive coronary stenosis, abnormal PET-MPI semiquantitative parameters, reduced CT-derived fractional flow reserve (CT-FFR), and a higher burden of high-risk plaque features (all *P* < 0.05). The total number of high-risk plaque features was significantly associated with the risk of MACEs (HR 1.898, 95% CI 1.015–3.546; *P* = 0.045) and showed a trend toward a linear relationship with event risk. PET-MPI semiquantitative parameters improved prognostic performance beyond the clinical baseline model (*P* = 0.011), and further incorporation of high-risk plaque feature count significantly improved model performance across functional models (all *P* < 0.05).

**Conclusions:**

AI-driven CCTA integrated with PET/CT dynamic MPI may provide prognostic information in patients with CAD. PET-MPI semiquantitative parameters and the total number of high-risk plaque features were associated with risk and may offer complementary and incremental value for predicting MACEs. These findings warrant further validation in larger cohorts.

## Introduction

Coronary artery disease (CAD) remains a leading cause of cardiovascular morbidity and mortality worldwide. Although advancements in pharmacotherapy and interventional techniques have led to enhanced clinical outcomes for CAD patients [1], the early detection of individuals at high risk for future major adverse cardiovascular events and optimizing personalized therapeutic approaches continue to represent major clinical challenges [2].

In the noninvasive diagnostic assessment of CAD, positron emission tomography (PET) myocardial perfusion imaging (MPI) is widely regarded as the “gold standard” for detecting myocardial ischemia. Dynamic PET-MPI enables both semiquantitative assessment of relative myocardial perfusion and absolute quantification of myocardial blood flow (MBF) and coronary flow reserve (CFR). These critical quantitative parameters offer a comprehensive insight into epicardial coronary artery function and microvascular function [3, 4]. Prior studies have consistently demonstrated a robust link between the severity of myocardial ischemia and quantitative flow metrics derived from PET-MPI and patient outcomes [5, 6].

Coronary computed tomography angiography (CCTA) is a widely used noninvasive imaging technique, enabling direct visualization of coronary artery anatomy, luminal stenosis and atherosclerotic plaque. However, conventional visual assessment of CCTA is largely focused on luminal stenosis and is subject to interobserver variability, providing limited quantitative information regarding plaque burden and composition. Recent advances in artificial intelligence (AI)-based CCTA analysis have enabled automated, reproducible, and comprehensive quantitative assessment of coronary stenosis, plaque burden, plaque composition, and high-risk plaque (HRP) features. Furthermore, by leveraging extensive anatomical data and computational fluid dynamics algorithms, these tools facilitate the calculation of CT-derived fractional flow reserve (CT-FFR), thereby delivering a crucial functional hemodynamic assessment of stenotic lesions [7, 8]. Notably, the SCOT-HEART trial [9] demonstrated the clinical value of incorporating CCTA into the diagnostic pathway of patients with suspected CAD and highlighted the importance of anatomical plaque assessment for risk stratification. Together, these advances suggest that AI-enhanced CCTA may provide additional quantitative information for cardiovascular risk assessment beyond conventional visual interpretation.

Existing studies have demonstrated the prognostic value of CCTA-derived stenosis severity, CT-FFR, HRP features, and PET-MPI parameters individually. However, whether AI-derived quantitative plaque characteristics provide incremental prognostic information beyond functional imaging and whether integrated anatomical-functional assessment can improve risk stratification remain insufficiently investigated. Consequently, this study integrates AI-based CCTA analysis with dynamic PET/CT MPI, leveraging multimodal and multiparametric anatomical and functional information to evaluate their combined ability to predict major adverse cardiovascular events (MACEs) in patients with suspected or confirmed CAD and to determine the incremental prognostic value of this integrated approach.

## Materials and methods

### Study population

**Fig. 1:**
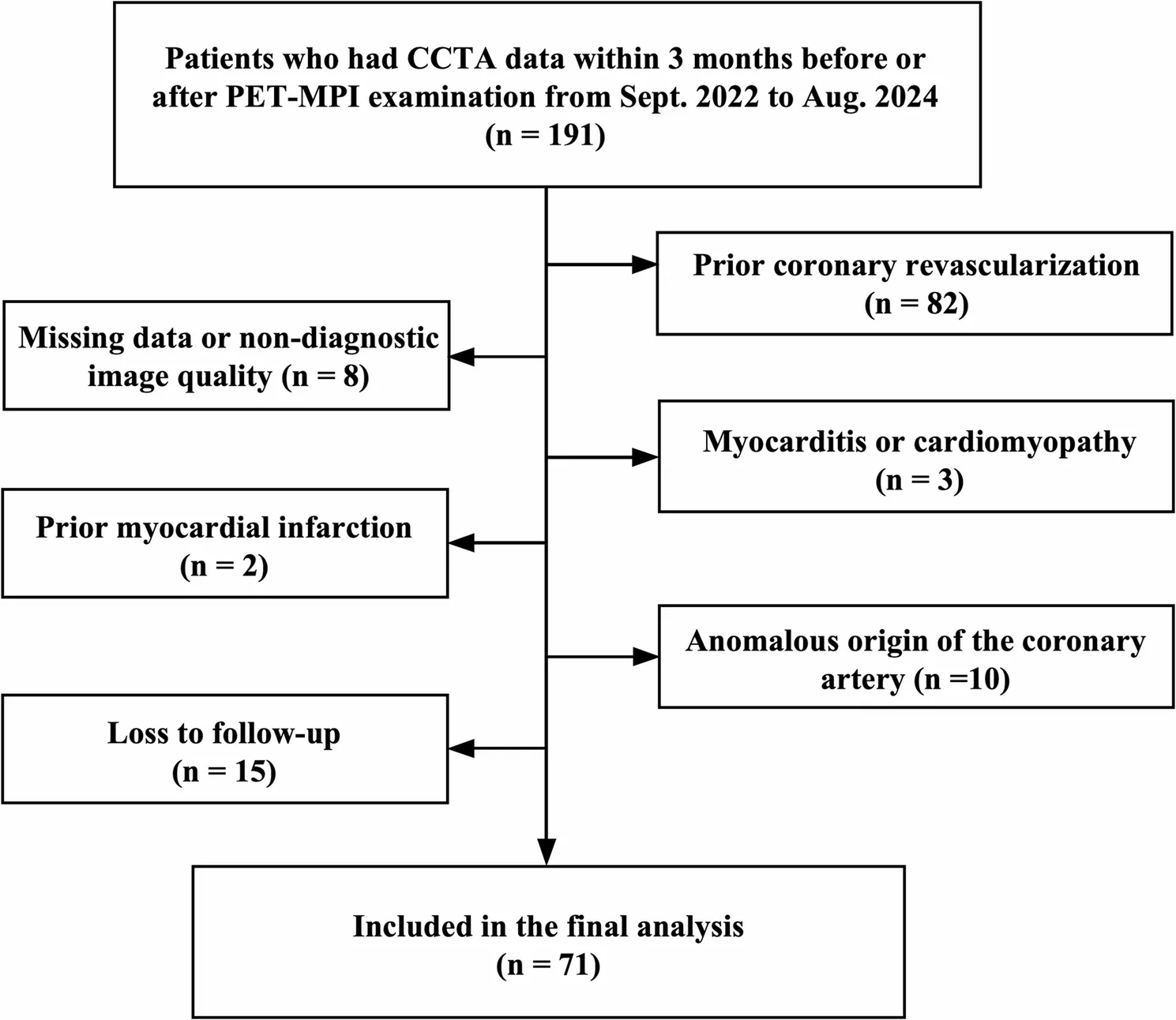
Flowchart of patient inclusion. CCTA, coronary computed tomography angiography; PET, positron emission tomography; MPI, myocardial perfusion imaging

Patients with suspected or confirmed CAD who underwent dynamic PET/CT MPI at TEDA International Cardiovascular Hospital between September 2022 and August 2024 were retrospectively and consecutively enrolled. All patients received at least 12 months of follow-up. Patients with suspected or confirmed CAD were identified according to the clinical assessment of the treating cardiologists. Patients were referred for PET/CT MPI and CCTA as part of routine clinical evaluation and management. The inclusion criteria comprised: (1) clinically confirmed or suspected CAD, (2) CCTA data acquired within 3 months before or after dynamic PET-MPI examination, with no intervening coronary revascularization, and (3) no contraindications to adenosine stress or intolerance of the dynamic PET-MPI acquisition process. Conversely, the exclusion criteria encompassed: (1) documented prior myocardial infarction; (2) any history of coronary revascularization; (3) suspected or confirmed myocarditis, cardiomyopathy, or valvular heart disease; (4) anomalous coronary artery origin; and (5) non-diagnostic image quality that precluded reliable image interpretation or quantitative analysis, missing data, or loss to follow-up. Patient baseline characteristics—including sex, age, body mass index (BMI), medical history, smoking history, alcohol consumption, and family history—were systematically collected via an electronic medical record information system. Ultimately, 71 patients, including 42 males (59.2%) and 29 females (40.8%) aged 34–80 years (mean age 60.17 ± 12.36), were enrolled (Fig. 1). This study was conducted in accordance with the Declaration of Helsinki and its subsequent amendments and was approved by the Ethics Committee of TEDA International Cardiovascular Hospital (No.2026-0109-2). Informed consent was obtained from all patients.

### PET/CT MPI

Prior to PET/CT MPI, all patients discontinued taking relevant cardiovascular medications, including vasoactive drugs, beta-blockers, calcium antagonists, nitrates, theophyllines, nicorandil, and dipyridamole. Additionally, they avoided consumption of tea, coffee, or any caffeine-containing beverages for at least 24 h. The imaging agent ^13^N-NH_3_·H_2_O was manufactured by our medical cyclotron (MINItrace Qilin, GE HealthCare, Chicago, IL, USA), while PET/CT imaging was performed using a Discovery Elite system (NM690; GE HealthCare, Waukesha, WI, USA). One-day rest/adenosine stress dynamic imaging was employed, ensuring an interval of at least 40 min between the two injections. Intravenous access was pre-established in both upper arms, connected to a three-way stopcock. After the CT scan precisely localized the target region, low-dose CT attenuation correction scanning was subsequently carried out (using a tube voltage of 120 kV and tube current of 20 mA). For stress imaging, adenosine was intravenously infused at a rate of 0.14 mg/kg/min for 6 min according to body weight, and heart rate and blood pressure were continuously monitored throughout the procedure. PET acquisition commenced 10–15 s prior to the injection of the imaging agent. A bolus of 370–555 MBq (10–15 mCi) 13 N-NH3·H2O was administered was administered either at rest or at the third minute of adenosine infusion during stress imaging, immediately followed by a 10-mL saline flush. The dynamic PET acquisition was performed using the following framing sequence: 10 s/frame × 12 frames; 30 s/frame × 2 frames; 60 s/frame × 1 frame; and 360 s/frame × 1 frame. The rest or stress-gated tomography acquisition—acquiring 8 frames per cardiac cycle for 8 min—was performed after completing the dynamic scan. All PET images were reconstructed using a three-dimensional iterative reconstruction algorithm with corrections for attenuation, scatter, random events, geometric efficiency, and spatial resolution. PET/CT registration was visually verified before quantitative analysis and manually adjusted when necessary to ensure accurate image alignment and quantitative measurements. Upon completion, all acquired data were exported to the Q.PET workstation (Cedars-Sinai Medical Center, Los Angeles, CA, USA) for processing by a physician with more than 3 years of experience in cardiac PET imaging. Global left ventricular (LV) MBF measurements at both rest and stress states were automatically quantified using the Q.PET software based on a validated two-tissue three-compartment kinetic model for 13 N-NH3 dynamic imaging. From these values, we derived the global CFR, calculated as stress MBF divided by rest MBF. In addition, horizontal long-axis, vertical long-axis, and short-axis images of the left ventricle were reconstructed. These images served as the basis for deriving semiquantitative global left ventricular parameters, including the summed stress score (SSS), the summed rest score (SRS), and the summed difference score (SDS), where SDS equals SSS minus SRS.

### CCTA

The scanning equipment was a Revolution 256 - row wide - body detector CT scanner (GE Healthcare). CCTA scans were obtained via the prospective electrocardiogram gating protocol, with the following scan parameters: a tube voltage of 100 or 120 kV according to body mass index, automated tube current modulation (400–740 mA), a slice thickness of 0.625 mm, a collimator width of 16 cm, a matrix of 512 × 512, a display field of view (FOV) of 25 cm, an adaptive statistical iterative reconstruction - V iterative algorithm of 50%. Prior to CCTA acquisition, the heart rate was maintained below 75 beats/min whenever feasible. Iohexol contrast medium (350 mg iodine/mL) was injected via an antecubital vein using a dual-head power injector (Ulrich Medical, Ulm, Germany), with a contrast volume of 60–70 mL and an injection rate of 4.5–5.0 mL/s, followed by a saline flush. After scanning, the device automatically calculated and reconstructed the optimal phase. If the optimal phase was unsatisfactory, multiple phase reconstructions were manually performed at 5% intervals to select the optimal phase.

After the optimal phase images were obtained, all CCTA raw data were transferred to a quantitative analysis software workstation (uAI Discover-CCTA, CTA Coronary Intelligence Analysis System, United Imaging Healthcare, Shanghai, China). A specially trained diagnostician used this workstation to perform CCTA-based quantitative coronary analysis (QCA) of the most stenotic lesion in each vessel.

CT-FFR computation was performed using a computational fluid dynamics (CFD)-based software platform (uCT-FFR version 1.5, United Imaging Healthcare). This software first simulates hemodynamic parameters across the entire coronary tree using CFD and then applies a deep learning model trained on the CFD-derived data to estimate CT-FFR values [10].

### Coronary artery feature analysis from CCTA

After obtaining the reconstructed CCTA coronary images, quantitative and qualitative analyses of coronary artery characteristics were performed using a dedicated workstation (uAI Discover-CCTA, CTA Coronary Intelligence Analysis System, United Imaging Healthcare, Shanghai, China). The software automatically identified the inner and outer vessel contours and, based on predefined CT attenuation thresholds for plaque components, generated quantitative measurements including total plaque burden (TPB), calcified plaque burden (CPB), non-calcified plaque burden (NCPB), lipid plaque burden (LPB), fibrous-fatty plaque burden (FFPB), and fibrotic plaque burden (FPB). Plaque composition was classified according to predefined CT attenuation thresholds: lipid plaque (< 30 HU), fibrous-fatty plaque (30–130 HU), fibrotic plaque (131–350 HU), and calcified plaque (> 350 HU). High-risk plaque (HRP) features were qualitatively assessed, including positive remodeling (PR), low-attenuation plaque (LAP), the napkin-ring sign (NRS), and spotty calcification (SC). PR was defined as a remodeling index > 1.1. LAP was defined as plaque with attenuation < 30 HU. NRS was defined as a central low-attenuation plaque core surrounded by a ring-like higher attenuation rim. SC was defined as focal calcification < 3 mm in diameter within a plaque. HRP features were assessed at the patient level. A patient was considered positive for a given HRP feature if the feature was present in any coronary plaque. For the calculation of the total HRP feature count, each occurrence of PR, LAP, NRS, and SC was counted separately, and the total number of HRP features was obtained by summing all occurrences across all coronary plaques within an individual patient.

### Criteria for classification

PET-MPI: (1) Patients were categorized based on global left ventricular PET-CFR, defining reduced CFR as a PET-CFR value < 2.5 [11, 12], which divided them into a normal CFR group and a reduced CFR group. (2) Semiquantitative abnormalities were defined as a global LV-SSS ≥ 4 or LV-SDS ≥ 2 [13, 14]. Patients were subsequently stratified into a group with normal semiquantitative parameters and a group with abnormal semiquantitative parameters.

CCTA: QCA–derived stenosis ≥ 50% in any coronary vessel defined obstructive coronary stenosis [15] at the case level, dividing them into non-obstructive and obstructive stenosis groups. Furthermore, CT-FFR ≤ 0.80 in any coronary vessel served as the case-level criterion for ischemia-causing stenosis [16, 17]; patients were consequently stratified into a normal CT-FFR group and a reduced CT-FFR group.

### Patient follow-up

Patient follow-up for MACEs was conducted by reviewing electronic medical records from our institution and/or through telephone interviews. The date of PET/CT MPI was predefined as the index date for follow-up analyses. The median follow-up duration after PET/CT dynamic MPI was 25.69 months (IQR 21.83–29.55).

MACEs were defined as the occurrence of at least one of the following endpoints: all-cause death, nonfatal myocardial infarction, new-onset heart failure, rehospitalization for angina, or late coronary revascularization (including percutaneous coronary intervention or coronary artery bypass grafting), with the time to first event used for analysis [18, 19]. Myocardial infarction was defined as an elevation of cardiac troponin above the 99th percentile upper reference limit with a dynamic change, accompanied by evidence of acute myocardial ischemia, including typical ischemic symptoms, new ischemic ECG changes, or imaging evidence of new regional wall-motion abnormalities [20]. Follow-up duration was defined as the interval from the date of the PET/CT dynamic MPI examination to the occurrence of MACEs or the last follow-up date.

### Statistical analysis

Based on follow-up outcomes, patients were categorized into a non-MACEs group and a MACEs group. All statistical analyses were performed using IBM SPSS version 29.0 (IBM Corp., Armonk, NY, USA) and RStudio version 2025.05.1 + 513 (Posit Software, Boston, MA, USA).

Continuous variables with a normal distribution were expressed as mean ± standard deviation and compared between groups using independent-samples t-tests. Nonnormally distributed continuous variables were expressed as the median and interquartile range and were compared using the Mann–Whitney U test. Categorical variables were expressed as the frequency and composition ratio (%), and group comparisons were conducted using the chi-square test; when necessary, the continuity correction chi-square test or Fisher’s exact test was applied.

Before conducting multivariable Cox regression analyses, LASSO regression was used to select candidate variables in order to reduce multicollinearity and minimize the risk of model overfitting. Ten-fold cross-validation was performed to determine the optimal penalty parameter (λ), and variables with non-zero coefficients were retained for further analysis. These variables were subsequently entered into multivariable Cox regression analyses to evaluate their independent associations with the risk of MACEs, with results expressed as hazard ratio (HR) and 95% confidence intervals (95% CIs). The proportional hazards assumption of the Cox regression model was evaluated using Schoenfeld residuals. For continuous variables that remained statistically significant in the multivariable Cox models, restricted cubic spline (RCS) analyses were performed to evaluate dose–response relationships with the risk of MACEs. RCS models were constructed using four knots located at the recommended percentiles of the distribution of the analyzed variable.

To evaluate the incremental prognostic value of multimodal imaging parameters for MACEs, a hierarchical Cox proportional hazards modeling strategy was applied. First, a clinical risk factor–based model was established as the baseline model. Multiple large-scale multicenter studies have consistently demonstrated that age and diabetes are robust independent predictors of CAD and future MACEs [21–25]; therefore, age and diabetes were included a priori in the baseline model (Model 1). Subsequently, functional imaging parameters were added to Model 1 to construct functional models (Model 2) and assess their prognostic contribution. The functional parameters evaluated in this study included CFR (Model 2a), semiquantitative parameters (Model 2b), and CT-FFR (Model 2c). Finally, variables selected through LASSO regression and further evaluated in multivariable Cox regression analyses were individually incorporated into each functional model to generate extended models (Model 3a, Model 3b, Model 3c). Model performance was compared using the likelihood ratio test (LRT). Harrell’s C-index was calculated to assess model discrimination, and internal validation was performed using bootstrap resampling (B = 1000) to obtain optimism-corrected estimates. The Akaike information criterion (AIC) was used to evaluate model fit.

All statistical analyses were considered statistically significant at *P* < 0.05.

## Results

### Baseline clinical characteristics and comparison between groups

A total of 71 patients were enrolled in this study (mean age 60.17 ± 12.36 years; 59.2% male). The median interval between CCTA and PET/CT MPI was 2 days (IQR, 1–5 days). CCTA was performed before PET/CT MPI in 68 patients (95.8%) and after PET/CT MPI in 3 patients (4.2%). None of the latter patients experienced MACEs during follow-up. The median follow-up duration after PET/CT dynamic MPI was 25.69 months (IQR 21.83–29.55). Eighteen patients (18/71, 25.4%) experienced MACEs during follow-up, including 2 all - cause deaths (2/71, 2.8%), 3 late coronary revascularizations (3/71, 4.2%), and 13 rehospitalizations for angina (13/71, 18.3%). No cases of nonfatal myocardial infarction or new - onset heart failure occurred during the follow - up period.

The baseline clinical characteristics of the two groups are presented in Table 1. The proportions of patients with obstructive coronary stenosis, abnormal semiquantitative parameters, and reduced CT-FFR were significantly higher in the MACEs group than in the non-MACEs group (all *P* < 0.05). No significant differences were observed between the two groups regarding other baseline characteristics, clinical risk factors, or the prevalence of reduced CFR (all *P* > 0.05).

**Table 1:** Comparison of clinical characteristics between non-MACEs and MACEs groups

Clinical Characteristics	Non-MACEs (*n* = 53)	MACEs (*n* = 18)	*P*
Sex (male)	32(60.4%)	10(55.6%)	0.719
Age	59.06 ± 12.373	63.44 ± 12.045	0.195
BMI	25.915 ± 3.490	25.183 ± 3.228	0.437
Hypertension	28(52.8%)	14(77.8%)	0.063
Hyperlipidemia	13(24.5%)	5(27.8%)	0.784
Diabetes mellitus	12(22.6%)	3(16.7%)	0.592
Smoking history	17(32.1%)	5(27.8%)	0.733
Alcohol consumption history	5(9.4%)	2(11.1%)	0.837
Family history	5(9.3%)	5(27.8%)	0.053
Obstructive coronary stenosis	28(52.8%)	16(88.9%)	0.006*
Reduced CFR	25(47.2%)	8(44.4%)	0.841
Abnormal semiquantitative parameters	18(34.0%)	13(72.2%)	0.005*
Reduced CT-FFR	12(22.6%)	10(55.6%)	0.009*

*P < 0.05; BMI: body mass index; CFR: coronary flow reserve; CT-FFR: CT-derived fractional flow reserve

### AI-CCTA coronary imaging characteristics and comparison between groups

Compared with the non-MACEs group, patients in the MACEs group had significantly higher TPB, NCPB, LPB, FFPB, and FPB. Additionally, the prevalence of HRP features—including PR, NRS, and SC—was significantly higher in the MACEs group, and the total number of HRP features was also greater (all *P* < 0.05). No significant differences were found between the two groups in CPB and LAP (both *P* > 0.05) (Table 2).

**Table 2:** Comparison of AI-CCTA coronary imaging characteristics between non-MACEs and MACEs groups

coronary imaging characteristics	Non-MACEs (*n* = 53)	MACEs (*n* = 18)	*P*
TPB	27.90(4.83,46.92)	63.54(46.25,101.62)	< 0.001*
CPB	3.21(0.05,11.98)	3.13(1.33,31.75)	0.158
NCPB	22.95(3.98,35.77)	57.93(37.49,68.11)	< 0.001*
LPB	2.03(0.07,4.24)	5.11(2.53,8.79)	< 0.001*
FFPB	8.88(0.65,16.05)	23.82(13.49,30.10)	< 0.001*
FPB	9.06(1.86,16.24)	23.31(14.55,34.97)	0.001*
PR	40(75.5%)	18(100.0%)	0.049*
LAP	42(79.2%)	18(100.0%)	0.084
NRS	17(32.1%)	12(66.7%)	0.010*
SC	27(50.9%)	14(77.8%)	0.046*
The total number of HRP features	4.0(2.0,7.5)	11.5(7.0,15.0)	< 0.001*

*TPB* Total plaque burden, *CPB* Calcified plaque burden, *NCPB* Non-calcified plaque burden, *LPB* Lipid plaque burden, *FFPB* Fibrous-fatty plaque burden, *FPB* Fibrotic plaque burden, *PR* Positive remodeling, *LAP* Low-attenuation plaque, *NRS* Napkin-ring sign, *SC* Spotty calcification, *HRP* High-risk plaque

*P < 0.05

Although PR and LAP were present in all patients who experienced MACEs, only PR demonstrated a significant between-group difference.

### Prognostic value of combined CCTA and PET-MPI parameters

**Fig. 2:**
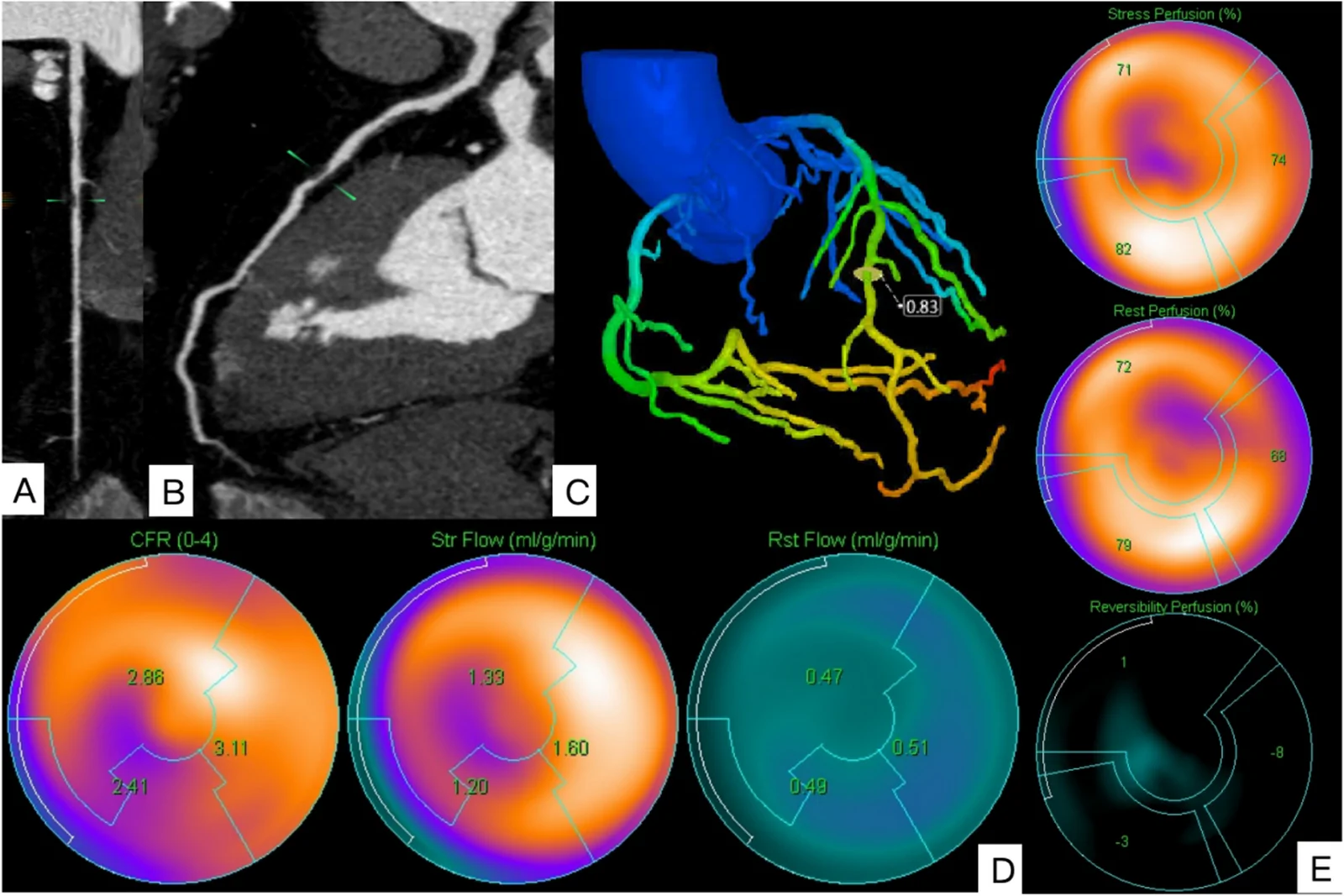
Representative case of multimodal and multiparametric evaluation of CAD using AI-CCTA combined with PET/CT dynamic MPI. A 44-year-old male patient. **A**, **B** CCTA revealed a non-calcified plaque that caused localized moderate stenosis in the LAD (QCA = 63%, green marker). **C** CT-FFR derived from CCTA showed a minimum CT-FFR value of 0.83, indicating no hemodynamically significant lesion. **D** Quantitative PET/CT MPI demonstrated normal CFR at both the patient and vessel levels (LV-CFR = 2.84). **E** The PET/CT MPI polar map revealed a small area of reversible ischemia in the LAD territory, which was consistent with semiquantitative abnormalities (SSS = 5, SDS = 2). During follow-up, the patient was rehospitalized for angina. In addition, AI-CCTA analysis identified a markedly elevated total number of HRP features (*n* = 13). This case illustrates that, even in the absence of overt functional impairment suggested by CFR or CT-FFR, PET-MPI semiquantitative parameters and the total number of HRP features may provide critical prognostic information and play an important role in identifying potentially high-risk individuals. CAD: coronary artery disease; AI: artificial intelligence; CCTA: coronary computed tomography angiography; PET: positron emission tomography/computed tomography; MPI: myocardial perfusion imaging; LAD: left anterior descending artery; QCA: quantitative coronary analysis; CT-FFR: computed tomography–derived fractional flow reserve; LV: left ventricular; CFR: coronary flow reserve; SSS: summed stress score; SDS: summed difference score

All candidate variables presented in Tables 1 and 2, including clinical characteristics, PET-MPI parameters, CCTA findings, and AI-CCTA-derived plaque characteristics, were initially entered into the LASSO regression model for variable selection. After preliminary screening, two variables—FFPB and the total number of HRP features—were retained. These variables were subsequently included in a multivariable Cox regression analysis. The results revealed that the total number of HRP features (per 1-feature increase) was significantly associated with the risk of MACEs (HR: 1.898, 95% CI: 1.015–3.546, *P* = 0.045), whereas FFPB showed no significant association with the occurrence of MACEs (HR: 1.310, 95% CI: 0.802–2.139, *P* = 0.281). The proportional hazards assumption was assessed using Schoenfeld residuals, and no significant violations were observed for either variable (all *P* > 0.05) or for the overall model (global *P* = 0.228), indicating that the proportional hazards assumption was satisfied. (A representative case is shown in Fig. 2).

**Fig. 3:**
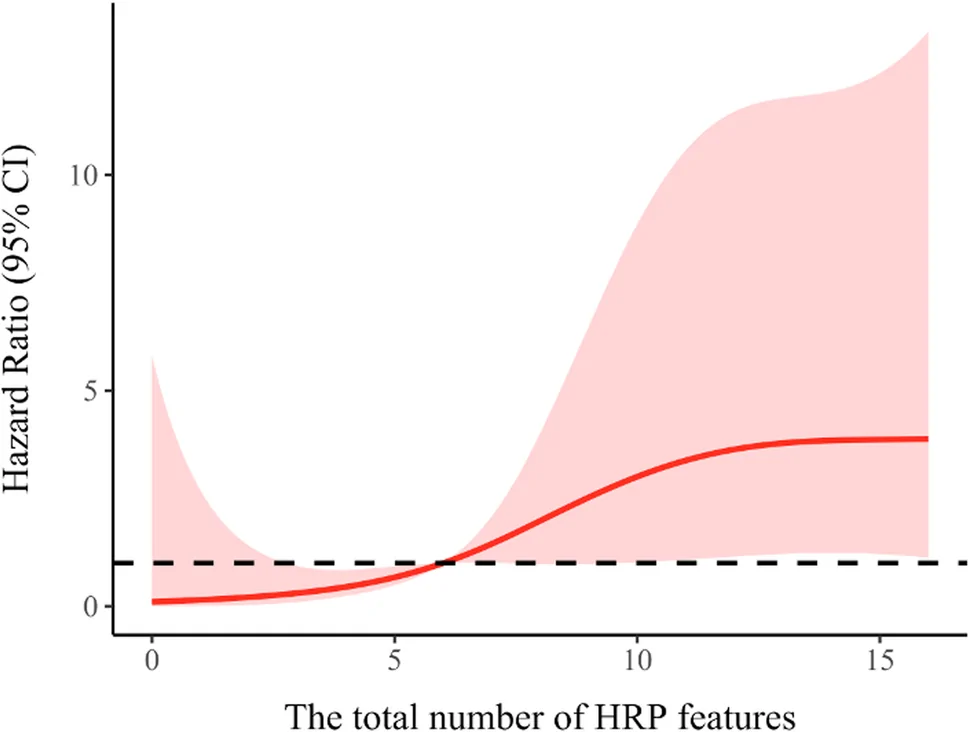
Restricted cubic spline (RCS) analysis of the association between the total number of high-risk plaque (HRP) features and the risk of MACEs

In addition, the total number of HRP features was incorporated into an RCS model to further evaluate its dose–response relationship with the risk of MACEs. The RCS analysis demonstrated a trend toward a linear association between the total number of HRP features and the risk of MACEs (P _overall_ = 0.012, P _non−linear_ = 0.263), indicating that the risk of MACEs increased steadily as the number of HRP features increased (Fig. 3).

### Incremental prognostic value of multimodal imaging parameters based on AI-CCTA combined with PET-MPI (Table 3; Fig. 4)

In the baseline clinical model (Model 1) constructed using age and diabetes, the incorporation of different functional parameters resulted in varying model performance. When PET-MPI semiquantitative parameters (normal vs. abnormal) were used as the functional reference standard, the addition of semiquantitative parameters to Model 1 (Model 2b) was associated with improved model fit compared with the baseline model (*P* = 0.011). In contrast, when PET-CFR or CT-FFR were used as functional reference standards, the addition of PET-CFR (Model 2a) or CT-FFR (Model 2c) to Model 1 was not associated with a significant change in model performance (both *P* > 0.05).

**Table 3:** Comparison of the incremental prognostic value of Models 1–3 based on three functional reference standards

	Model 1	Model 2	Model 3
Using CFR as the functional standard (a)			
-2 LOG likelihood	140.75	140.36	126.43
Chi-square change (change vs. previous model)	Reference	+ 0.39	+ 13.93
P (change vs. previous model)	-	0.532	< 0.001
Harrell’s C-index	0.460	0.428	0.676
(95% CI)	(0.246–0.721)	(0.235–0.708)	(0.479–0.904)
AIC	144.75	146.36	134.43
Using semiquantitative parameters as the functional standard (b)			
-2 LOG likelihood	140.75	134.319	123.718
Chi-square change (change vs. previous model)	Reference	+ 6.433	+ 10.601
P (change vs. previous model)	-	0.011	0.001
Harrell’s C-index	0.460	0.572	0.680
(95% CI)	(0.246–0.721)	(0.380–0.800)	(0.510–0.891)
AIC	144.75	140.319	131.718
Using CT-FFR as the functional standard (c)			
-2 LOG likelihood	140.75	136.95	127.85
Chi-square change (change vs. previous model)	Reference	+ 3.800	+ 9.1
P (change vs. previous model)	-	0.051	0.003
Harrell’s C-index	0.460	0.480	0.663
(95% CI)	(0.246–0.721)	(0.321–0.753)	(0.465–0.870)
AIC	144.75	142.952	135.846

Model 1 represents the baseline model including age and diabetes;

Model 2 represents the models constructed by adding functional parameters (CFR, PET-MPI semiquantitative parameters, or CT-FFR) to Model 1;

Model 3 represents the extended models obtained by further incorporating the total number of high-risk plaque features into Model 2;

*AIC* Akaike information criterion

**Fig. 4:**
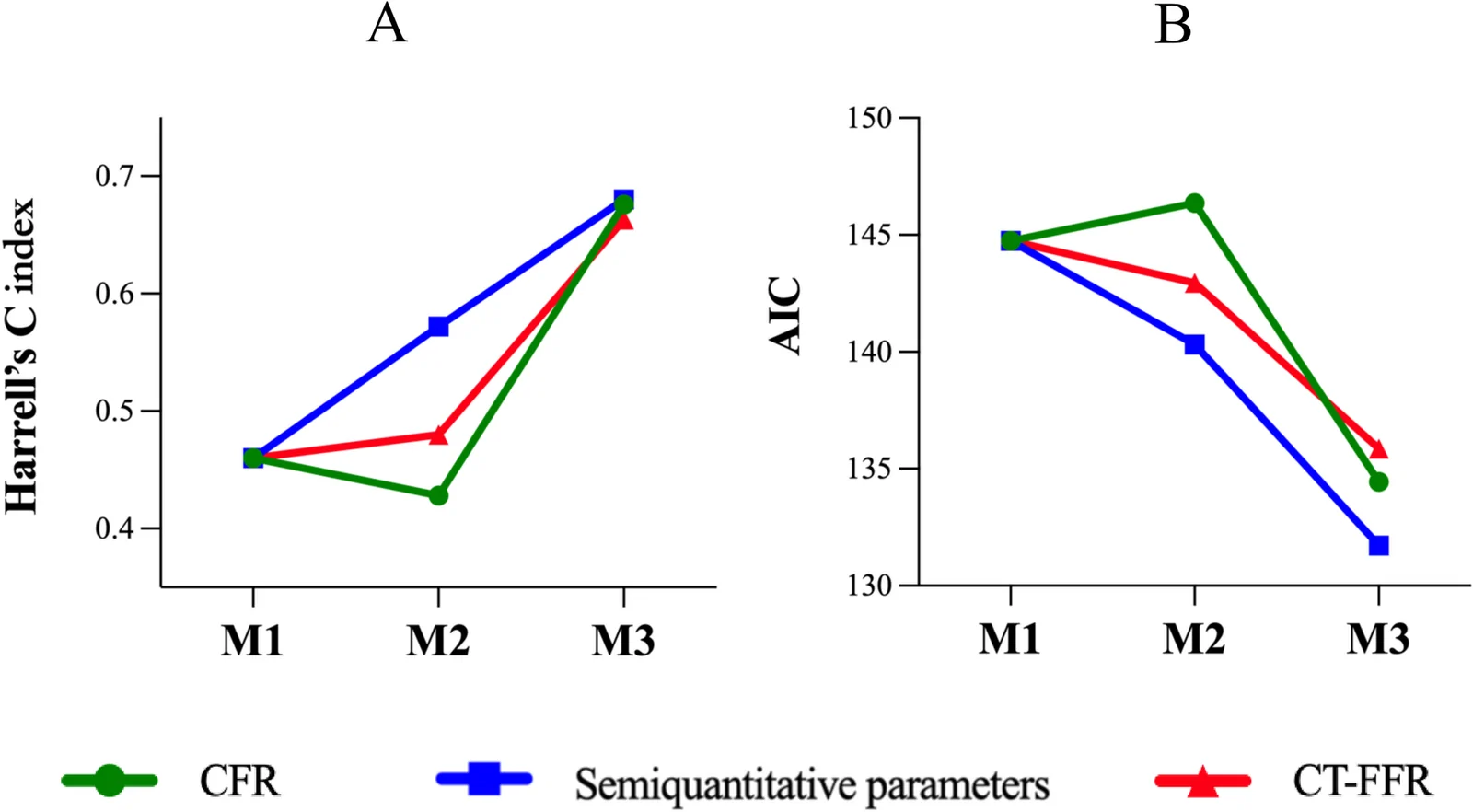
Incremental prognostic value of multimodal imaging parameters from AI-CCTA combined with PET-MPI for predicting MACEs. **A** shows the trend in Harrell’s C-index from Model 1 to Model 3, with higher Harrell’s C-index values indicating better model discrimination and stronger predictive performance. **B** illustrates the trend in AIC across the three models, where lower AIC values indicate better model fit and greater model parsimony. Model 1 represents the baseline model, which includes age and diabetes. Model 2 represents the models constructed by adding functional parameters (CFR, PET- MPI semiquantitative parameters, or CT-FFR) to Model 1. Model 3 represents the extended models obtained by further incorporating the total number of high - risk plaque features into Model 2. AI: artificial intelligence; CCTA: coronary computed tomography angiography; PET: positron emission tomography; MPI: myocardial perfusion imaging; MACEs: major adverse cardiovascular events; AIC: Akaike information criterion; CFR: coronary flow reserve; CT-FFR: computed tomography–derived fractional flow reserve

Further incorporation of the total number of high-risk plaque features into each functional model (Models 3a–3c) was associated with improved model fit across all functional pathways (all *P* < 0.05), accompanied by a decrease in AIC, suggesting improved goodness of fit. In addition, Harrell’s C-index increased to a range of 0.663–0.680, suggesting modest but improved discrimination after adding the total number of high-risk plaque features (Fig. 4). 

## Discussion

This study evaluated the prognostic and incremental prognostic value of combining AI-CCTA-derived coronary imaging characteristics with functional parameters from PET/CT dynamic MPI in predicting MACEs among patients with CAD. The results showed that the proportion of patients with obstructive coronary stenosis, abnormal PET-MPI semiquantitative parameters, and reduced CT-FFR was significantly higher in the MACEs group than in the non-MACEs group. This finding is consistent with previous research results [19, 26–28]. In contrast, the prevalence of reduced CFR did not differ significantly between the two groups. These results suggest that patients presenting with obstructive stenosis, abnormal semiquantitative parameters, or reduced CT-FFR were more frequently observed among patients who experienced MACEs, and that the occurrence of adverse events is more strongly influenced by focal obstructive lesions and associated hemodynamic impairment. Regarding AI-CCTA-derived coronary imaging characteristics, the MACEs group demonstrated a greater overall plaque burden and a higher concentration of HRP features. These findings indicate that the development of MACEs may be associated not only with focal anatomical stenosis but also closely with the overall plaque burden and plaque vulnerability.

After exploratory variable selection using LASSO regression and subsequent multivariable Cox proportional hazards analysis, the present study found that the total number of HRP features was associated with the risk of MACEs (HR: 1.898, 95% CI: 1.015–3.546, *P* = 0.045). Moreover, a trend toward a linear association was observed between the total count of high-risk plaque features and the risk of MACEs (P _overall_ = 0.012, P _non − linear_ = 0.263). This finding suggests that the risk of adverse events increases progressively with the accumulation of high-risk plaque characteristics, rather than demonstrating a single threshold effect. It is also noteworthy that among the HRP phenotypes, PR and LAP showed the highest prevalence in the MACEs group. Consistent with our findings, previous studies [26, 29, 30] have confirmed the association between HRP features and the occurrence of MACEs, with particularly pronounced predictive value in women and younger individuals. Among these markers, LAP has been highlighted as one of the most powerful predictors of future MACEs. However, the present study suggests that the cumulative burden of HRP features, rather than any individual HRP phenotype, may provide more comprehensive prognostic information for risk stratification in patients with CAD.

Regarding the incremental prognostic value of multimodal imaging parameters for predicting MACEs, incorporating PET-MPI semiquantitative parameters (Model 2b) into the baseline model constructed from clinical risk factors (age and diabetes; Model 1) was associated with improved model fit and discrimination. In contrast, adding CFR (Model 2a) or CT-FFR (Model 2c) did not show a significant improvement in model performance.

Furthermore, when the total number of high-risk plaque features was further incorporated into each functional model (Models 2a–2c) to construct Models 3a–3c, the model fit and prognostic performance improved consistently across all functional standards. This improvement was accompanied by a reduction in AIC and an increase in Harrell’s C-index, suggesting modest but consistent improvements in prognostic performance following the addition of high-risk plaque burden.

Several studies [13, 31, 32] have reported that abnormal semiquantitative perfusion is closely associated with clinical outcomes in patients with CAD, as these measures directly reflect the degree of myocardial perfusion impairment—one of the fundamental pathophysiological drivers of MACEs. Myocardial perfusion status is strongly linked to ischemic symptoms and the need for revascularization, thereby enabling direct risk stratification and providing quantitative metrics for prognostic assessment. In contrast, CFR, as an integrated index of global coronary flow reserve, is easily influenced by multiple factors such as age, metabolic conditions, and diffuse microvascular dysfunction. Because CFR is derived from the ratio of stress to rest MBF, both of which may decline simultaneously, the ratio may remain within the normal range despite true perfusion impairment. This may lead to false-negative cases in which CFR appears preserved while perfusion is abnormal. In addition, the follow-up duration in the present study was relatively short, and the endpoint events primarily consisted of rehospitalization for angina and late revascularization. Prior investigations [33, 34] have demonstrated that CFR exhibits stronger predictive value for “hard” endpoints, such as death or myocardial infarction, particularly in studies with longer follow-up periods, whereas its predictive ability for “soft” endpoints—such as recurrent angina or late revascularization—is limited. Therefore, the predominance of soft endpoints in the present study may have attenuated the observed prognostic contribution of certain functional parameters, particularly CFR. Our findings are consistent with this pattern. The advantage of CT-FFR lies predominantly in evaluating the functional significance of stenosis in individual vessels. However, when summarized at the patient level, the lesion-specific information may be diluted by inter-vessel heterogeneity, resulting in limited incremental value once age and diabetes are incorporated into the baseline clinical model. In comparison, semiquantitative perfusion parameters can more directly capture key information closely linked to symptoms, ischemia, and revascularization, which may partly explain their stronger association with the predominantly symptom-driven composite endpoint observed in the present study [35, 36].

Furthermore, this study suggests that the total number of HRP features, rather than any single high-risk plaque feature, may better reflect the overall burden of vulnerable plaques and is associated with the risk of MACEs. In addition, we observed that regardless of the functional pathway adopted, incorporating the total HRP feature count consistently enhanced model fit and modestly improved the predictive performance for MACEs. These results suggest that this parameter was associated with the risk of MACEs and may offer incremental prognostic value in this exploratory cohort. This finding underscores the complementary nature of anatomical and functional information. Integrating functional indices together with the total number of HRP features into the risk model enables the construction of a more comprehensive “anatomy–function–prognosis” evaluation framework. Such an approach facilitates quantitative and integrated risk assessment, and may contribute to more comprehensive risk stratification in patients with CAD. Our results further suggest that relying solely on functional parameters in clinical practice may not sufficiently identify all high-risk individuals. Additional assessment of the total HRP feature count may further improve prognostic evaluation and help identify patients at elevated risk. Previous studies have suggested that for patients with a high burden of vulnerable plaques, intensification of secondary prevention, optimization of pharmacologic therapy, and regular imaging follow-up may be considered [37]. A combined anatomical–functional evaluation may provide complementary information for future risk assessment. With ongoing advances in artificial intelligence and quantitative imaging analysis, integrated AI-guided multimodal assessment may further facilitate individualized risk assessment and support future research on precision imaging in CAD.

This study also has several limitations. First, this was a single-center investigation with a relatively small sample size, a limited number of MACEs—particularly hard cardiovascular events—and a relatively short follow-up duration, which may have limited the generalizability of the findings and affected the stability of the prognostic models. In addition, because both PET/CT MPI and CCTA findings were available to the treating physicians, subsequent clinical management, particularly rehospitalization and late revascularization, may have been influenced by the imaging results. Furthermore, several potentially important clinical confounders, including symptoms, formal pretest probability, imaging indications, baseline medication, and treatment changes after imaging, were not systematically available in this retrospective study and therefore could not be incorporated into the analyses. Consequently, residual confounding cannot be excluded, and the observed associations should be interpreted with caution. Second, because only patients who completed both PET/CT MPI and CCTA within the predefined time interval were eligible for inclusion, potential selection bias toward a relatively healthier cohort cannot be completely excluded. Third, external validation was lacking in both model selection and model construction, which may affect the robustness of the proposed models. Although LASSO regression and bootstrap resampling were applied to reduce model complexity and enhance internal model stability, a residual risk of overfitting cannot be completely excluded because of the limited number of events. Fourth, the functional parameters (CFR, PET-MPI semiquantitative parameters, and CT-FFR) were analyzed in a dichotomized form, which may have resulted in a loss of information and an underestimation of their true effects. Finally, CCTA-derived coronary imaging features may vary depending on scanner type, acquisition conditions, and analytical software, potentially introducing heterogeneity across studies and limiting the generalizability of imaging-based measurements. Therefore, the conclusions of this study should be further validated using multicenter, large-sample datasets with standardized imaging analysis protocols.

## Conclusions

In summary, the combined utilization of AI-based CCTA and PET/CT dynamic MPI shows significant clinical value in prognostic assessment for patients with CAD. PET-MPI semiquantitative parameters can improve the prediction of MACEs when incorporated into the clinical baseline model. Additionally, the total number of HRP features was associated with the risk of MACEs and may provide incremental prognostic value across different functional models. These findings emphasize its potential usefulness in risk assessment and management of CAD, offering strong imaging evidence to support accurate prognostication and individualized therapeutic strategies. 

## Data Availability

The datasets used and/or analysed during the current study are available from the corresponding author on reasonable request.

## References

[1] Roth GA, Mensah GA, Johnson CO, Addolorato G, Ammirati E, Baddour LM, et al. Global burden of cardiovascular diseases and risk factors, 1990–2019: update from the GBD 2019 study. J Am Coll Cardiol. 2020;76(25):2982–3021. 10.1016/j.jacc.2020.11.010 PMC7755038 33309175

[2] Vrints C, Andreotti F, Koskinas KC, Rossello X, Adamo M, Ainslie J, et al. 2024 ESC Guidelines for the management of chronic coronary syndromes. Eur Heart J. 2024;45(36):3415–537. 10.1093/eurheartj/ehae177 39210710

[3] Schindler TH, Fearon WF, Pelletier-Galarneau M, Ambrosio G, Sechtem U, Ruddy TD, et al. Myocardial perfusion PET for the detection and reporting of coronary microvascular dysfunction: a JACC: cardiovascular imaging expert panel statement. JACC Cardiovasc Imaging. 2023;16(4):536–48. 10.1016/j.jcmg.2022.12.015 36881418

[4] El-Tallawi KC, Aljizeeri A, Nabi F, Al-Mallah MH. Myocardial perfusion imaging using positron emission tomography. Methodist Debakey Cardiovasc J. 2020;16(2):114–21. 10.14797/mdcj-16-2-114 PMC7350808 32670471

[5] Kato T, Momose M, Uemura Y, Naya M, Matsumoto N, Hida S, et al. Association of the extent of myocardial ischemia with outcomes in patients with suspected coronary artery disease in Japan. J Cardiol. 2022;80(5):475–81. 10.1016/j.jjcc.2022.06.012 35835641

[6] Monroy-Gonzalez AG, Tio RA, de Groot JC, Boersma HH, Prakken NH, De Jongste MJL, et al. Long-term prognostic value of quantitative myocardial perfusion in patients with chest pain and normal coronary arteries. J Nucl Cardiol. 2019;26(6):1844–52. 10.1007/s12350-018-1448-8 PMC6908551 30288680

[7] Schulze K, Stantien AM, Williams MC, Vassiliou VS, Giannopoulos AA, Nieman K et al. Coronary CT angiography evaluation with artificial intelligence for individualized medical treatment of atherosclerosis: a Consensus Statement from the QCI Study Group. Nat Rev Cardiol. 2026;23:100–15. 10.1038/s41569-025-01191-6 40751112

[8] Baeßler B, Götz M, Antoniades C, Heidenreich JF, Leiner T, Beer M. Artificial intelligence in coronary computed tomography angiography: demands and solutions from a clinical perspective. Front Cardiovasc Med. 2023;10:1120361. 10.3389/fcvm.2023.1120361 PMC9978503 36873406

[9] CT coronary angiography in. patients with suspected angina due to coronary heart disease (SCOT-HEART): an open-label, parallel-group, multicentre trial. Lancet. 2015;385(9985):2383–91. 10.1016/S0140-6736(15)60291-4 25788230

[10] Tang CX, Liu CY, Lu MJ, Schoepf UJ, Tesche C, Bayer RR 2, et al. CT FFR for Ischemia-Specific CAD with a new computational fluid dynamics algorithm: a Chinese multicenter study. JACC Cardiovasc Imaging. 2020;13(4):980–90. 10.1016/j.jcmg.2019.06.018 31422138

[11] Zimmerli A, Salihu A, Antiochos P, Lu H, Pitta Gros B, Berger A, et al. Evolution of Coronary Microvascular Dysfunction Prevalence over Time and Across Diagnostic Modalities in Patients with ANOCA: A Systematic Review. J Clin Med. 2025;14(3):829. 10.3390/jcm14030829 PMC11818762 39941504

[12] Meeder JG, Hartzema-Meijer MJ, Jansen TPJ, Konst RE, Damman P, Elias-Smale SE. Outpatient management of patients with angina with no obstructive coronary arteries: how to come to a proper diagnosis and therapy. Front Cardiovasc Med. 2021;8:716319. 10.3389/fcvm.2021.716319 PMC8592903 34796207

[13] Caobelli F, Haaf P, Haenny G, Pfisterer M, Zellweger MJ. Prognostic value of myocardial perfusion scintigraphy in asymptomatic patients with diabetes mellitus at high cardiovascular risk: 5-year follow-up of the prospective multicenter BARDOT trial. Eur J Nucl Med Mol Imaging. 2021;48(11):3512–21. 10.1007/s00259-021-05349-5 PMC8440314 33884468

[14] Xiao F, Zhang W, He YN, Yang J, Liu X, Wang L, et al. Impaired perfusion in the myocardial microcirculation in asymptomatic patients with Stages 1–4 chronic kidney disease with intrarenal arterial lesions. Nephrol Dial Transpl. 2021;36(11):2066–75. 10.1093/ndt/gfaa245 33877359

[15] Miller RJH, Gransar H, Rozanski A, Dey D, Al-Mallah M, Chow BJW, et al. Simplified approach to predicting obstructive coronary disease with integration of coronary calcium: development and external validation. J Am Heart Assoc. 2023;12(24):e031601. 10.1161/JAHA.123.031601 PMC10863788 38108259

[16] Hecht HS, Narula J, Fearon WF. Fractional flow reserve and coronary computed tomographic angiography: a review and critical analysis. Circ Res. 2016;119(2):300–16. 10.1161/CIRCRESAHA.116.307914 27390333

[17] Feng Y, Xu Z, Zhang L, Zhang Y, Xu H, Zhuang X, et al. Machine-learning-derived radiomics signature of pericoronary tissue in coronary CT angiography associates with functional ischemia. Front Physiol. 2022;13:980996. 10.3389/fphys.2022.980996 PMC9550214 36225300

[18] Liu M, Zhen Y, Shang J, Dang Y, Zhang Q, Ni W, et al. The predictive value of lesion-specific pericoronary fat attenuation index for major adverse cardiovascular events in patients with type 2 diabetes. Cardiovasc Diabetol. 2024;23(1):191. 10.1186/s12933-024-02272-5 PMC11149297 38835028

[19] Guo B, Jiang M, Guo X, Tang C, Zhong J, Lu M, et al. Diagnostic and prognostic performance of artificial intelligence-based fully-automated on-site CT-FFR in patients with CAD. Sci Bull (Beijing). 2024;69(10):1472–85. 10.1016/j.scib.2024.03.053 38637226

[20] Thygesen K, Alpert JS, Jaffe AS, Chaitman BR, Bax JJ, Morrow DA, et al. Fourth Universal Definition of Myocardial Infarction (2018). Circulation. 2018;138(20):e618–51.. 10.1161/CIR.0000000000000617 30571511

[21] Blankstein R, Budoff MJ, Shaw LJ, Goff DC Jr., Polak JF, Lima J, et al. Predictors of coronary heart disease events among asymptomatic persons with low low-density lipoprotein cholesterol MESA (Multi-Ethnic Study of Atherosclerosis). J Am Coll Cardiol. 2011;58(4):364–74. 10.1016/j.jacc.2011.01.055 21757113

[22] Jalali A, Hassanzadeh A, Najafi MS, Nayebirad S, Dashtkoohi M, Karimi Z, et al. Predictors of major adverse cardiac and cerebrovascular events after percutaneous coronary intervention in older adults: a systematic review and meta-analysis. BMC Geriatr. 2024;24(1):337. 10.1186/s12877-024-04896-4 PMC11015672 38609875

[23] Hippisley-Cox J, Coupland C, Brindle P. Development and validation of QRISK3 risk prediction algorithms to estimate future risk of cardiovascular disease: prospective cohort study. BMJ. 2017;357:j2099. 10.1136/bmj.j2099 PMC5441081 28536104

[24] Parasca CA, Head SJ, Milojevic M, Mack MJ, Serruys PW, Morice MC, et al. Incidence, characteristics, predictors, and outcomes of repeat revascularization after percutaneous coronary intervention and coronary artery bypass grafting: the SYNTAX trial at 5 years. JACC Cardiovasc Interv. 2016;9(24):2493–507. 10.1016/j.jcin.2016.09.044 28007201

[25] SCORE2 risk prediction. algorithms: new models to estimate 10-year risk of cardiovascular disease in Europe. Eur Heart J. 2021;42(25):2439–54. 10.1093/eurheartj/ehab309 PMC8248998 34120177

[26] Feuchtner G, Kerber J, Burghard P, Dichtl W, Friedrich G, Bonaros N, et al. The high-risk criteria low-attenuation plaque < 60 HU and the napkin-ring sign are the most powerful predictors of MACE: a long-term follow-up study. Eur Heart J Cardiovasc Imaging. 2017;18(7):772–9. 10.1093/ehjci/jew167 27502292

[27] Yoda S, Nakanishi K, Tano A, Hori Y, Suzuki Y, Matsumoto N, et al. Major cardiac event risk scores estimated with gated myocardial perfusion imaging in Japanese patients with coronary artery disease. J Cardiol. 2016;67(1):64–70. 10.1016/j.jjcc.2015.04.008 25982667

[28] Driessen RS, Raijmakers PG, Stuijfzand WJ, Knaapen P. Myocardial perfusion imaging with PET. Int J Cardiovasc Imaging. 2017;33(7):1021–31. 10.1007/s10554-017-1084-4 PMC5489578 28188475

[29] Ferencik M, Mayrhofer T, Bittner DO, Emami H, Puchner SB, Lu MT, et al. Use of high-risk coronary atherosclerotic plaque detection for risk stratification of patients with stable chest pain: a secondary analysis of the PROMISE randomized clinical trial. JAMA Cardiol. 2018;3(2):144–52. 10.1001/jamacardio.2017.4973 PMC5838601 29322167

[30] Williams MC, Moss AJ, Dweck M, Adamson PD, Alam S, Hunter A, et al. Coronary artery plaque characteristics associated with adverse outcomes in the SCOT-HEART study. J Am Coll Cardiol. 2019;73(3):291–301. 10.1016/j.jacc.2018.10.066 PMC6342893 30678759

[31] Bourque JM, Beller GA. Stress myocardial perfusion imaging for assessing prognosis: an update. JACC Cardiovasc Imaging. 2011;4(12):1305–19. 10.1016/j.jcmg.2011.10.003 22172788

[32] Assante R, Zampella E, Cantoni V, Green R, D’Antonio A, Mannarino T, et al. Prognostic value of myocardial perfusion imaging by cadmium zinc telluride single-photon emission computed tomography in patients with suspected or known coronary artery disease: a systematic review and meta-analysis. Eur J Nucl Med Mol Imaging. 2023;50(12):3647–58. 10.1007/s00259-023-06344-8 PMC10547640 37480369

[33] Souza A, Rosenthal MH, Moura FA, Divakaran S, Osborne MT, Hainer J, et al. Body composition, coronary microvascular dysfunction, and future risk of cardiovascular events including heart failure. JACC Cardiovasc Imaging. 2024;17(2):179–91. 10.1016/j.jcmg.2023.07.014 PMC10922555 37768241

[34] Sciagrà R, Passeri A, Bucerius J, Verberne HJ, Slart RH, Lindner O, et al. Clinical use of quantitative cardiac perfusion PET: rationale, modalities and possible indications. position paper of the cardiovascular committee of the European association of nuclear medicine (EANM). Eur J Nucl Med Mol Imaging. 2016;43(8):1530–45. 10.1007/s00259-016-3317-5 26846913

[35] Kogame N, Ono M, Kawashima H, Tomaniak M, Hara H, Leipsic J, et al. The impact of coronary physiology on contemporary clinical decision making. JACC Cardiovasc Interv. 2020;13(14):1617–38. 10.1016/j.jcin.2020.04.040 32703589

[36] Shaw LJ, Blankstein R, Brown DL, Dhruva SS, Douglas PS, Genders TSS, et al. Controversies in diagnostic imaging of patients with suspected stable and acute chest pain syndromes. JACC Cardiovasc Imaging. 2019;12(7 Pt 1):1254–78. 10.1016/j.jcmg.2019.05.009 31272608

[37] Dimitriadis K, Pyrpyris N, Theofilis P, Mantzouranis E, Beneki E, Kostakis P, et al. Computed tomography angiography identified high-risk coronary plaques: from diagnosis to prognosis and future management. Diagnostics (Basel). 2024;14(15):1671. 10.3390/diagnostics14151671 PMC11311283 39125547

